# We Need You: Influence of Hiring Demand and Modified Applicant Testing on the Physical Fitness of Law Enforcement Recruits

**DOI:** 10.3390/ijerph17207512

**Published:** 2020-10-15

**Authors:** Robert G. Lockie, J. Jay Dawes, Matthew R. Moreno, Megan B. McGuire, Tomas J. Ruvalcaba, Ashley M. Bloodgood, Joseph M. Dulla, Robin M. Orr

**Affiliations:** 1 Department of Kinesiology, California State University, Fullerton, CA 92835, USA; moreno.matthewr@csu.fullerton.edu (M.R.M.); megan_mcguire@csu.fullerton.edu (M.B.M.); tjruvalcaba@csu.fullerton.edu (T.J.R.); abloodgood17@csu.fullerton.edu (A.M.B.); 2 School of Kinesiology, Applied Health and Recreation, Oklahoma State University, Stillwater, OK 74078, USA; jay.dawes@okstate.edu; 3 Tactical Research Unit, Bond University, Robina, QLD 4229, Australia; joseph.dulla@student.bond.edu.au (J.M.D.); rorr@bond.edu.au (R.M.O.)

**Keywords:** 75 yard pursuit run, aerobic fitness, multistage fitness test, occupational testing, police, tactical

## Abstract

A challenge for law enforcement agencies is the many positions that need filling. Agencies may modify their applicant test battery (ATB; multilevelled testing including fitness, background checks, psychological evaluations) to increase the hiring pool of potential recruits by augmenting the utility of testing. This study determined fitness differences of law enforcement recruits hired under two different ATB protocols. Retrospective analysis was conducted on seven academy classes (442 males, 84 females) hired under an older ATB, and one class (45 males, 13 females) hired under a newer ATB. Recruits completed the following before academy: 60 s push-ups and sit-ups (muscular endurance); vertical jump (lower-body power); medicine ball throw (upper-body power); 75 yard pursuit run (75PR; change-of-direction speed); and 20 m multistage fitness test (20MSFT; aerobic fitness). Independent sample t-tests (*p* ≤ 0.001) and effect sizes (*d*) evaluated between-group fitness differences for recruits hired under the different ATB protocols (combined sexes, males, and females). There were no significant differences between the ATB groups. However, newer ATB female recruits completed 13% fewer 20MSFT shuttles than the older ATB group, which, although not significant (*p* = 0.007), did have a moderate effect (*d* = 0.62). Females hired under the newer ATB had lower aerobic fitness, which could impact physical training performance and graduation.

## 1. Introduction

Law enforcement can be a physically demanding profession. During a shift, officers can be required to exert force during pushing, pulling, lifting, carrying, or dragging tasks [1]. Officers may also need to complete job-specific tasks, including driving vehicles [2], defensive tactics [3], civilian or partner rescue [4], and pursuing and apprehending suspects [3,4]. Due to these demands, physical training forms an important component of the academy training process. Academy training is where law enforcement training staff will develop recruits to meet the physical challenges of the job, while also teaching the necessary procedures, skills, and expected values and behavior expected of a law enforcement officer [5,6].

The hiring process for a law enforcement agency (LEA) is multilayered, and can include (but is not limited to) fitness and medical examinations, background checks, and psychological evaluations [7,8]. Many LEAs use physical fitness testing as part of the hiring process in an attempt to ensure recruits have the underlying capacities needed to complete academy training [9,10,11] and future job-specific tasks [12]. For example, recruits with a better grip strength [3] and vertical jump (VJ) [13] were less likely to experience injuries and illness. Dawes et al. [9] found that the number of push-ups completed in 60 s and VJ height were the best predictors of recruit graduation from a state patrol academy. Shusko et al. [11] detailed that push-ups completed in 60 s and aerobic fitness measured by the 2.4 km (1.5 mile run) were the best predictors of graduation for municipal police academy recruits. In their study, Lockie et al. [10] documented that recruits who separated due to injury or physical training failures were slower in the 75 yard pursuit run (75PR) and completed fewer 20 m multistage fitness test (20MSFT) shuttles. Ensuring recruits have the fitness necessary to complete academy training is important. Losing recruits during academy can bring high financial costs to an agency [11], and recruiting individuals with greater fitness could alleviate this risk.

A challenge for many LEAs is that they have a high number of positions that need to be filled. Finding enough suitable candidates is a problem faced by law enforcement organizations [14]. There are a number of societal issues that are affecting the number of suitable applicants an agency will receive. For example, within the American general population, the number of males and females who are physically active has gone down [15,16], which has coincided with an increase in obesity across almost all adult age groups [17,18]. This means that there are likely to be less people in the general population who could meet the minimum fitness standards required for many agencies. In addition, there are also people who view law enforcement as a less attractive profession [19,20], further diluting the available candidate pool.

Because of the need to fill positions, some agencies may adapt their hiring process to potentially allow more recruits to reach academy training [21,22]. Agencies may also review hiring practices as required by local and federal laws, consent decrees, evolving job standards, and pertinent research. The LEA in this study modified their applicant test battery (ATB), which incorporated multiple levels of testing including fitness, background checks, and psychological evaluations, to increase the number of applicants eligible to attend the academy. This allowed for the training of 100–200 more recruits per year. Information about all changes to ATB procedures were not made available to the researchers. However, specific to fitness testing, the 2.4 km run was replaced by the 20MSFT and the arm ergometer was removed from hiring fitness tests. This was in part changed to allow for greater utility in testing, such that multiple sites could be used to widen the candidate pool. The purpose of this study was to determine any differences in the fitness of law enforcement recruits hired under the two different ATB protocols from one LEA. It was hypothesized that there would be minor, if any, differences in the fitness of recruits hired under the older and newer ATB.

## 2. Materials and Methods

### 2.1. Subjects

Data were released with consent from one USA-based LEA. A total of 584 recruits (age = 27.31 ± 6.19 years; height = 1.73 ± 0.09 m; body mass = 80.50 ± 13.60 kg) were analyzed in this study, comprising 487 males (age = 27.21 ± 5.93 years; height = 1.75 ± 0.07 m; body mass = 83.65 ± 12.06 kg) and 97 females (age = 27.81 ± 7.39 years; height = 1.63 ± 0.06 m; body mass = 64.66 ± 9.11 kg). The sample included 526 recruits (442 males, 84 females) that were hired under the older ATB, and 58 recruits (45 males, 13 females) hired under the newer ATB. Only those recruits with full datasets were considered in the analysis for this study. The characteristics of the subjects in this study, and the between-sex ratio, was similar to that from previous law enforcement research [4,6,10,12,23,24,25,26]. Based on the archival nature of this study, the institutional ethics committee approved the use of pre-existing data (HSR-17-18-370). This study also conformed to the Declaration of Helsinki recommendations [27].

### 2.2. Procedures

Retrospective analysis was conducted on seven academy classes hired under an older ATB from 2017–2018, and one class hired under the newer ATB in 2019. The data in this study were collected by staff in the week preceding academy training for all the classes that were analyzed, and established procedures were used. Recruits had familiarity with the assessments used at the start of academy, as they were required to complete tests such as the push-ups, sit-ups, and the 75PR as part of the hiring process. Nonetheless, the data collected by the staff were used for record at the LEA. The staff were all trained by a certified tactical strength and conditioning facilitator who verified the proficiency of each staff member. Prior to testing, each recruit’s age, height, and body mass were recorded. Height was measured using a portable stadiometer (seca, Hamburg, Germany), while body mass was recorded by electronic digital scales (Omron Healthcare, Kyoto, Japan). Following this, recruits from all classes completed a series of dynamic movements (e.g., squats, lunges, push-ups, shoulder and hip mobility movements) that served as a warm-up. These movements were an extension of the standardized stretches used by this agency as part of the testing within the hiring process [28]. All tests were conducted outdoors on concrete or asphalt surfaces at the LEA’s training facility on a day scheduled by the LEA staff. Testing occurred between 09:00–14:00 (9:00 a.m.–2:00 p.m.) depending on recruit availability. Recruits generally did not eat in the 2–3 h prior to their testing session as they were completing employee-specific documentation for the LEA. The weather conditions for testing for all classes were typical of the climate of southern California. Although conducting testing outdoors is not ideal, there was no available indoor testing facility available for this agency and these procedures followed standard guidelines for this LEA [4,6,10,12,23,24,29]. Recruits rotated through the fitness tests in small groups of 3–4, except for the 20MSFT which was completed last in groups of 14–16. Recruits were allocated to a testing station before rotating to the next station once all groups were completed. All recruits completed the 20MSFT last. The testing procedures were typical of staff from this agency, and within a range of published research [10,25,29,30,31]. The recruits were permitted to consume water as required during testing.

### 2.3. Push-Ups

Upper-body muscular endurance was assessed via a push-up test where recruits completed as many repetitions as possible in 60 s. This is a standard test in law enforcement, and the protocol for this test followed established procedures [4,6]. A tester placed a fist on the floor directly under the recruit’s chest to ensure they descended to an appropriate depth. Although there may be some limitations with this approach, this ensured recruits descended to the required depth. All female recruits were partnered with a female tester. On the start command, the tester began the stopwatch and the recruit flexed their elbows and lowered themselves until their chests contacted the tester’s fist before they extended their elbows to return to the start position. Recruits performed as many push-ups as possible using this technique within the time period. The recorded result was the number of correctly completed repetitions.

### 2.4. Sit-Ups

Abdominal muscular endurance was assessed via the sit-up test where recruits completed as many repetitions as possible in 60 s. The sit-up test is also a standard test in law enforcement [4,6]. The recruits laid on their backs with their knees flexed to 90°, heels flat on the ground, and arms crossed over the chest. The feet were held to the ground by a tester who also counted the repetitions as they were positioned in a manner in which they could view the technique and could communicate with the recruit. On the start command, recruits raised their shoulders from the ground while keeping their arms crossed over the chest and touched their elbows to their knees. The recruit then descended back down until their shoulder blades contacted the ground. Recruits completed as many repetitions as possible using this technique within the time period. The recorded result was the number of correctly completed repetitions.

### 2.5. Vertical Jump (VJ)

A Vertec apparatus (Perform Better, West Warwick, RI, USA) was used to measure the VJ height. The VJ provided an indirect measure of lower-body power, and established protocols were used to measure jump height [29]. The recruit stood side-on to the Vertec (on the dominant side), and while keeping their heels on the ground reached upward as high as possible to displace as many vanes as possible. The last vane moved was the zero reference. The recruit then jumped as high as possible, with no preparatory step, and height was recorded from highest vane moved. No restrictions were placed on the range of countermovement during the jump. Each subject completed two trials, with a between-trial recovery time of a minimum of 60 s. These procedures followed that recommended by the National Strength and Conditioning Association [32,33], and that from a multitude of previous studies [10,29,30,31,34,35,36,37]. VJ height was calculated in inches by subtracting the standing reach height from the jump height, before being converted to cm, with the best trial used for analysis.

### 2.6. The 75 Yard Pursuit Run (75PR)

**Figure 1:**
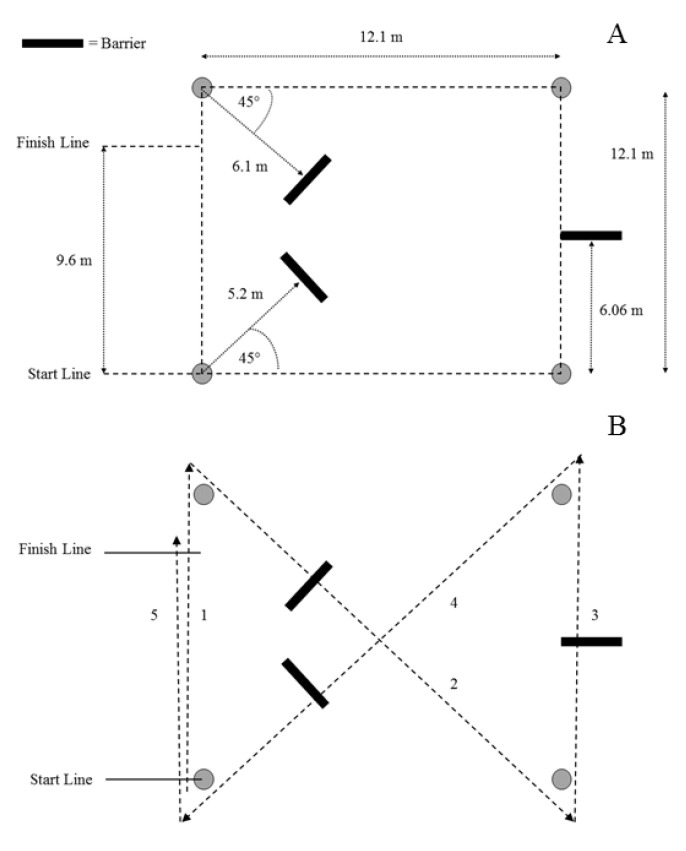
The 75PR dimensions in meters (m; (**A**)) and the running direction (numbered in order; (**B**)).

The 75PR was designed to simulate a foot pursuit for a law enforcement officer [12,37], and provided a measure of change-of-direction speed (Figure 1). The recruit completed five linear sprints about a square grid (each side was 12.1 m; the diagonal distance was 17.1 m), while completing four, 45° direction changes zig-zagging across the grid. Recruits stepped over three barriers that were 2.44 m in length and 0.15 m in height that simulated curbs during three of the five sprints. Time was recorded via a stopwatch, from the initiation of movement at the start, until the recruit crossed the finish line. Stopwatch timing was the standard measurement technique for this test [12]. Testers trained in the use of stopwatch timing procedures for running tests can record reliable data [38]. The procedures used in this study to measure the 75PR has been shown to have high trial-to-trial reliability (intra-class correlation coefficient = 0.85) [39]. Two trials were completed with at a minimum of 2 min rest between trials, due to how recruits rotated through this testing station. This also followed recommendations from the National Strength and Conditioning Association [32,33], and the 75PR data collection procedures have featured in numerous published studies [10,12,24,30,39,40]. The fastest trial, recorded in seconds, was analyzed.

### 2.7. Medicine Ball Throw (MBT)

The MBT was used to indirectly measure upper-body power, with established procedures utilized [29]. Recruits sat on the ground with their head, shoulders, and lower back against a concrete wall, and projected a 2 kg medicine ball (Sport Supply Group, Inc., Farmers Branch, TX, USA) as far as possible using a two-handed chest pass. The ball was lightly dusted with chalk to assist with grip, and to mark the landing spot of the ball. Throw distance was measured using a standard tape measure as the perpendicular distance from the wall to the chalk-marking closest to the wall made by the ball to the nearest centimeter. Two trials were completed, with a between-trial recovery time of a minimum of 60 s. As for the VJ, These procedures followed National Strength and Conditioning Association recommendations [32,33] previous research [10,29,30]. The best trial, reported in meters, was analyzed.

### 2.8. Multistage Fitness Test (20MSFT)

The 20MSFT was used to measure maximal aerobic capacity in the recruits and was conducted outdoors on an asphalt surface, which was the standard venue used at the LEA’s facility [23]. Recruits were required to run back and forth between two lines spaced 20 m apart, which were indicated by markers. The speed of running for this test was standardized by pre-recorded auditory cues (i.e., beeps) played from an iPad handheld device (Apple Inc., Cupertino, CA, USA) connected via Bluetooth to a portable speaker (ION Block Rocker, Cumberland, RI, USA). The speaker was located in the center of the running area, and positioned in such a way that it would not interfere with the recruits. The test was terminated when the recruit was unable to reach the lines twice in a row in accordance with the auditory cues. This test was scored according to the final level and stage the recruit was able to achieve, and the level and stage results were converted to the total number of shuttles completed. This approach has been used in a number of law enforcement-specific studies [9,10,23,26,30,41,42,43,44].

### 2.9. Statistical Analysis

All statistical analyses were computed using the Statistics Package for Social Sciences (Version 26.0; IBM Corporation, New York, NY, USA). Normality of the fitness test data was confirmed by visual analysis of the Q-Q plots [45,46,47]. Descriptive statistics (mean ± standard deviation (SD)) were calculated for each variable. Independent sample t-tests were utilized to calculate any differences in age, height, body mass, and physical fitness between the older and newer ATB groups. Data were analyzed with both sexes combined, in addition to males and females separately. The sexes were analyzed separately as numerous studies have documented sex differences in the physical performance of law enforcement populations [6,12,23,29]. Following Bonferroni correction for number of statistical tests of significance performed for the combined-sex, male, and female samples, the overall level of significance was set a priori at *p* ≤ 0.001 to limit type I errors [9]. Effect sizes (*d*) were also derived for the between-group comparisons for all recruits combined, males, and females, where the difference between the means was divided by the pooled SD [48]. A *d* less than 0.2 was considered a trivial effect; 0.2 to 0.6 a small effect; 0.6 to 1.2 a moderate effect; 1.2 to 2.0 a large effect; 2.0 to 4.0 a very large effect; and 4.0 and above an extremely large effect [49]. The effect size analyses were included in this study to ascertain how much difference existed between the groups irrespective of the *p* value [50,51]. This was also conducted to ensure the study results could be interpreted in a manner that would provide useful and practical information for law enforcement training staff and practitioners [9,10,25,51,52,53,54,55].

## 3. Results

The data for all recruits combined are shown in Table 1. For all recruits, equal variances were assumed for all variables except for body mass. There were no significant between-group differences in age, height, body mass, or any of the fitness tests. All effect sizes were trivial to small. Table 2 displays the data for the male recruits. For the males, equal variances were assumed for all variables except body mass and the MBT. There were no significant between-group differences for the males, and all comparisons had trivial-to-small effects.

Table 3 shows the female recruit data. For the females, equal variances were assumed for all variables except the 20MSFT. Female recruits hired under the newer ATB completed 16% fewer 20MSFT shuttles compared to females hired under the older ATB which, although not significant, had a moderate effect. There was also a moderate effect for the 3% faster 75PR completed by females hired under the newer ATB, although the differences with females from the older ATB was not significant. There were no other significant differences in age, height, body mass, or fitness test performance for the female recruits, with all effects trivial to small.

## 4. Discussion

This study investigated the characteristics and fitness test performance of law enforcement recruits hired under older and newer ATB. As stated, the LEA analyzed in this study in part changed their ATB procedures so they could more efficiently test more applicants in more locations to increase the pool eligible to attend the academy. The results from this study indicated that there were minimal differences between recruits hired under the older and newer ATB. However, the data did show that the females from the class hired under the newer ATB had lower aerobic capacity as measured by the 20MSFT. Given that many agencies want to hire and retain more women [56,57,58], this finding has important implications for LEA staff.

The data indicated that the characteristics (age, height, and body mass) of the recruits were similar between the older and newer ATB groups, were typical of similar populations from the literature [4,6,10,12,23]. When all recruits were combined, there was a non-significant, small effect in height for those hired under the older ATB compared to those hired under the newer ATB, which was likely due to the males. Indeed, the males in the older ATB group had a mean height taller than those in the newer ATB group. However, this difference, although it did have a small effect, was not significant. These results may have occurred due to the variation that occurs across recruits in different law enforcement academy classes [6]. Lockie et al. [6] found differences in the mean height of recruits across 11 classes from the one LEA. Nonetheless, it can be stated that the characteristics of the recruits hired under the newer ATB were not significantly different to those hired under the older ATB within the parameters of this study.

There were few differences in fitness test performance between the older and newer ATB groups. Each of the fitness tests included in this research has applicability to law enforcement recruits. Push-ups and sit-ups are staple tests of muscular endurance for law enforcement populations [4,6]. Greater muscular endurance measured by push-up repetitions could influence academy graduation [9,11], while better performance in both tests has been related to job tasks including running, jumping, and climbing [4]. The VJ provides a measure of lower-body power [29], and has been linked to academy graduation [9]. The MBT provides a measure of upper-body power [29], and this quality is needed in policing job tasks requiring upper-body pushing and striking [1]. Even with the newer ATB implemented to increase the number of recruits trained per year, this initial analysis of a class hired under these new procedures suggested that they were similar to established standards from the older ATB group relative to upper-body and abdominal muscular endurance, and upper- and lower-body power.

However, there were some differences between the older and newer ATB groups worth discussing. The most notable result from this study was the performance of the newer and older ATB female recruit groups in the 20MSFT. Female recruits hired under the older ATB were superior in the 20MSFT compared to those hired under the newer ATB. The disparity in completed shuttles was not significant in the context of this study, but the effect size difference was moderate. This is important to note, as the magnitude of difference shown by the effect size data arguably provides more important information to the practitioner than just the *p* value alone [51,59]. These results could be an area of focus for the newer ATB females for several reasons. Female recruits tend to demonstrate lower aerobic capacity measured by tests such as the 20MSFT and 2.4 km run compared to males [6,12,23]. Accordingly, many female recruits are starting academy at a physiological disadvantage compared to their male colleagues [6]. This could mean that females within an academy class are working at a higher relative intensity than their male counterparts, which could increase their risk of injury [60,61]. Further to this, better aerobic fitness has also been linked to greater potential for academy graduation [9,10,11]. and can also assist with recovery from exercise [62]. The ability to recover from physical training may indirectly help female recruits in other areas of academy (e.g., female recruits that can recover more effectively from an intense training session may be able to study the required academics more effectively). If female recruits being hired under a newer ATB consistently demonstrate lower aerobic fitness, this could lead to more females being separated from academy, due to factors such as physical training or academic failures or injury [10]. This could have large scale implications for the LEA relative to the retention of female recruits [56,57,58]. Several studies have noted the importance of targeted aerobic fitness training for female recruits [23], and that is supported by the results from this study. It should be noted that the sample of females in the newer ATB group was small (*n* = 13). Further analysis is required to determine whether females hired under the newer ATB consistently demonstrate lesser aerobic fitness. Additionally, the impact of any ATB changes on hiring numbers, whether this influences incoming female recruit fitness levels, and any potential impacts on recruit separation and/or injury rates should be a focus of future research.

The faster 75PR attained by females hired under the newer ATB compared to those from the older ATB had a moderate effect, although any differences were not significant in the context of this study. The 75PR was designed to simulate a foot pursuit and provides a measure of change-of-direction speed [12,37]. Lockie et al. [10] found that recruits who did not graduate from a law enforcement training academy tended to be slower in the 75PR. Accordingly, the 75PR provides a measure of physical characteristics important to law enforcement recruits. Post et al. [37] showed that greater linear and change-of-direction speed, and lower-body multidirectional power and isometric strength, correlated with a faster 75PR in male and female civilians. It could be that the newer ATB female recruits were superior in these qualities relative to the older ATB female recruits. However, only one class hired under the newer ATB was analyzed, and these data could have occurred due to the variation occurring across academy classes [6]. Further research is needed incorporating more classes hired under the newer ATB to determine whether these differences are consistent with future academy classes, and whether any differences arise in male recruits.

Several limitations to this study should be noted. There was a large discrepancy between the older and newer ATB groups (526 recruits vs. 58 recruits). Nevertheless, only data from one class hired under the newer ATB were available for the researchers. The researchers were not privy to all information regarding the newer ATB for a variety of reasons. As a result, this study did not consider other factors that could be influenced by newer ATB, including medical and psychological evaluations [7,8]. Nonetheless, it is of value to determine whether changes to an ATB are reflected in fitness tests across academy classes. The nature of field testing introduces some level of error to testing [33]. Nonetheless, the data collected and analyzed in this study were used for record at the LEA, and data collected via the procedures detailed have been published in numerous studies [4,6,10,12,23,24,26,29,30,31,39,40,41,42]. Maximal strength was not measured within the fitness testing battery, despite its importance for law enforcement job tasks [8,63,64]. This study also only included data from one LEA. As fitness test performance can vary across recruits from different agencies [65], individual LEAs may need to conduct their own studies to detail the effects of any changes their staff may make to their own ATB.

## 5. Conclusions

The results showed that there were limited fitness differences between classes hired under older and newer ATB from one agency. However, females in the recruit class hired under the newer ATB exhibited lower aerobic fitness measured by the 20MSFT. This could affect how the female recruits hired under the newer ATB perform during physical training, and influence their ability to graduate academy. Where possible, training staff should ensure that female recruits lacking in a specific physical quality receive appropriate training to develop shortcomings that could influence their ability to graduate academy. Future research should also investigate more classes hired under the newer ATB to determine whether the results shown in this study remain consistent with a larger sample.

## Figures and Tables

**Table 1:** Descriptive and fitness testing data from recruits hired under an older ATB (*n* = 526), versus one class hired under the LEA’s newer ATB (*n* = 58).

Variables	Older ATB	Newer ATB	*p*	*d*
Age (years)	27.29 ± 6.11	27.43 ± 6.48	0.874	0.02
Height (m)	1.74 ± 0.09	1.71 ± 0.09	0.018	0.33
Body mass (kg)	80.47 ± 13.25	80.75 ± 16.56	0.901	0.02
Push-ups (no.)	42.93 ± 14.44	42.45 ± 14.82	0.809	0.03
Sit-ups (no.)	35.68 ± 9.16	33.98 ± 7.43	0.173	0.20
VJ (cm)	53.52 ± 12.74	52.27 ± 15.90	0.492	0.09
MBT (m)	5.83 ± 1.21	5.96 ± 1.51	0.464	0.10
75PR (s)	17.08 ± 1.24	16.76 ± 1.02	0.057	0.28
20MSFT shuttles (no.)	54.37 ± 17.82	49.98 ± 14.07	0.070	0.27

**Table 2:** Descriptive and fitness testing data from male recruits hired under an older ATB (*n* = 442), versus one class hired under the LEA’s newer ATB (*n* = 45).

Variables	Older ATB	Newer ATB	*p*	*d*
Age (years)	27.16 ± 5.81	27.71 ± 7.01	0.550	0.09
Height (m)	1.76 ± 0.07	1.73 ± 0.08	0.021	0.40
Body mass (kg)	83.47 ± 11.70	85.44 ± 15.23	0.404	0.15
Push-ups (no.)	46.19 ± 12.65	47.00 ± 13.29	0.685	0.06
Sit-ups (no.)	36.44 ± 9.22	34.24 ± 7.77	0.123	0.26
VJ (cm)	55.91 ± 11.27	56.34 ± 15.53	0.818	0.03
MBT (m)	6.17 ± 0.95	6.50 ± 1.26	0.094	0.30
75PR (s)	16.83 ± 1.09	16.46 ± 0.85	0.029	0.38
20MSFT shuttles (no.)	55.36 ± 17.94	52.51 ± 14.56	0.302	0.17

**Table 3:** Descriptive and fitness testing data from female recruits hired under an older ATB (*n* = 84), versus one class hired under the LEA’s newer ATB (*n* = 13).

Variables	Older ATB	Newer ATB	*p*	*d*
Age (years)	28.02 ± 7.76	26.46 ± 4.29	0.481	0.25
Height (m)	1.62 ± 0.06	1.63 ± 0.05	0.839	0.18
Body mass (kg)	64.69 ± 9.14	64.53 ± 9.22	0.955	0.02
Push-ups (no.)	25.77 ± 10.70	26.69 ± 7.02	0.766	0.10
Sit-ups (no.)	31.68 ± 7.69	33.08 ± 6.30	0.535	0.20
VJ (cm)	40.89 ± 12.62	38.20 ± 6.25	0.453	0.27
MBT (m)	4.05 ± 0.78	4.08 ± 0.39	0.878	0.05
75PR (s)	18.42 ± 1.16	17.79 ± 0.89	0.064	0.61
20MSFT shuttles (no.)	49.13 ± 16.29	41.23 ± 7.56	0.007	0.62
